# Comparative Analysis of the Prevalence of Dysphagia in Patients with Mild COVID-19 and Those with Aspiration Pneumonia Alone: Findings of the Videofluoroscopic Swallowing Study

**DOI:** 10.3390/medicina59101851

**Published:** 2023-10-18

**Authors:** Junhyung Kim, Byungju Ryu, Yunhee Kim, Yireh Choi, Eunyoung Lee

**Affiliations:** Department of Physical Medicine and Rehabilitation, Sahmyook Medical Center, Seoul 02500, Republic of Korea; caumed2832@naver.com (J.K.); btjrbj@gmail.com (B.R.); yhkim76@hanmail.net (Y.K.);

**Keywords:** COVID-19, swallowing disorders, dysphagia, aspiration pneumonia, videofluoroscopic swallowing study

## Abstract

*Background and Objectives:* Patients recovering from mild coronavirus disease (COVID-19) reportedly have dysphagia or difficulty in swallowing. We compared the prevalence of dysphagia between patients diagnosed with mild COVID-19 and those diagnosed with aspiration pneumonia alone. *Materials and Methods:* A retrospective study was conducted from January 2020 to June 2023 in 160 patients referred for a videofluoroscopic swallowing study (VFSS) to assess for dysphagia. The cohort included 24 patients with mild COVID-19 and aspiration pneumonia, 30 with mild COVID-19 without aspiration pneumonia, and 106 with aspiration pneumonia alone. We reviewed the demographic data, comorbidities, and VFSS results using the penetration–aspiration scale (PAS) and functional dysphagia scale (FDS). *Results:* In a study comparing patients with mild COVID-19 (Group A) and those with aspiration pneumonia alone (Group B), no significant differences were observed in the baseline characteristics, including the prevalence of dysphagia-related comorbidities between the groups. Group A showed milder dysphagia, as evidenced by lower PAS and FDS scores, shorter oral and pharyngeal transit times (*p* = 0.001 and *p* = 0.003, respectively), and fewer residues in the vallecula and pyriform sinuses (*p* < 0.001 and *p* < 0.03, respectively). When Group A was subdivided into those with COVID-19 with (Group A1) and without aspiration pneumonia (Group A2), both subgroups outperformed Group B in terms of specific VFSS metrics, such as oral transit time (*p* = 0.01), pharyngeal transit time (*p* = 0.04 and *p* = 0.02, respectively), and residue in the vallecula (*p* = 0.04 and *p* = 0.02, respectively). However, Group B showed improved triggering of the pharyngeal swallowing reflex compared with Group A2 (*p* = 0.02). *Conclusion:* Mild COVID-19 patients showed less severe dysphagia than those with aspiration pneumonia alone. This finding was consistent across VFSS parameters, even when the COVID-19 group was subdivided based on the status of aspiration pneumonia.

## 1. Introduction

Since December 2019, coronavirus disease (COVID-19) has caused various medical problems worldwide. The atypical symptoms of COVID-19 include fever, cough, dyspnea, muscle pain, fatigue, occasional diarrhea, and vomiting [1]. The World Health Organization (WHO) classifies the severity of COVID-19 into four levels, mild, moderate, severe, and critical, based on the severity degree of clinical and respiratory symptoms, including increased oxygen demand [2]. In some patients with acute respiratory distress syndrome (ARDS), low oxygen saturation levels and inadequate oxygen delivery to organs may necessitate the need for invasive interventions, such as intubation or tracheotomy [3,4], and these interventions may increase the incidence of dysphagia [5,6]. Dysphagia is a medical condition characterized by difficulty in swallowing and poses significant challenges to patients’ nutritional intake and overall wellbeing [7]. Dysphagia can occur due to several factors, including neuromuscular conditions, structural abnormalities, esophageal motility anomaly, oropharyngeal dysfunction, and postoperative changes [8]. In particular, studies investigating COVID-19-related dysphagia have mainly focused on severely ill patients [9,10]. Approximately 60% of patients who are mechanically ventilated experience dysphagia even after extubation [11,12], and the risk of developing dysphagia increases as the duration of intubation and age increase [13]. In addition, patients with severe COVID-19 exhibit a higher degree of dysphagia than those with aspiration pneumonia [14].

However, the proportion of severe and critical cases of COVID-19 is considerably lower than that of mild-to-moderate cases [15]. Research on the incidence of dysphagia in patients with mild COVID-19 is scant. A study conducted by Can et al. [16] suggested that the risk of dysphagia in patients with mild COVID-19 should be considered. Shadi et al. [17] reported a high prevalence of self-reported swallowing difficulties among COVID-19 patients who were not intubated. Furthermore, studies comparing the characteristics of dysphagia in COVID-19 patients with mild-to-moderate symptoms and those with aspiration pneumonia are limited. Therefore, this study aimed to compare the swallowing function between patients with mild-to-moderate COVID-19, who comprise the majority of COVID-19 patients, and those with aspiration pneumonia. We formulated the hypothesis that patients with mild COVID-19 are likely to exhibit mild dysphagia.

## 2. Materials and Methods

### 2.1. Participants

**Figure 1:**
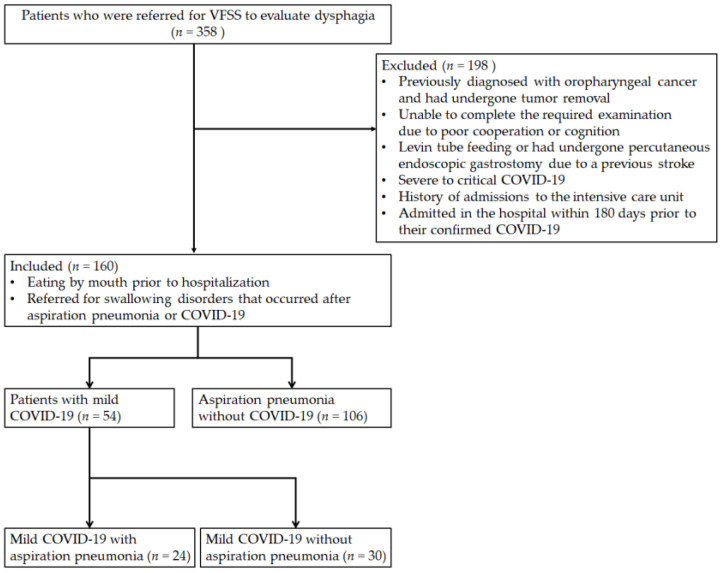
Flowchart of study participants.

We retrospectively collected the data of patients with dysphagia who were admitted to Sahmyook Medical Center from January 2020 to June 2023. A total of 358 patients were referred for a videofluoroscopic swallowing study (VFSS) to evaluate dysphagia. Patients who were (i) eating by mouth prior to hospitalization and (ii) referred for swallowing disorders that occurred after aspiration pneumonia or COVID-19 were included in the study. Patients who (i) were previously diagnosed with oropharyngeal cancer and had undergone tumor removal; (ii) were unable to complete the required examination due to poor cooperation or cognition; (iii) were on Levin tube feeding or had undergone percutaneous endoscopic gastrostomy due to a previous stroke; (iv) with severe to critical COVID-19; (v) with a history of admissions to the intensive care unit; and (vi) were admitted in the hospital within >180 days prior to their confirmed COVID-19 date were excluded. Only 160 patients were included in the final analysis. The patients were initially divided into two distinct groups: 54 patients with mild COVID-19 irrespective of pneumonia status (Group A) and 106 patients with aspiration pneumonia in the absence of COVID-19 (Group B). Additionally, patients with mild COVID-19 (Group A) were further subdivided into two groups based on the status of aspiration pneumonia to evaluate the variations in swallowing function across all subsets. Among these, 24 were diagnosed with mild COVID-19 aspiration pneumonia with dysphagia (Group A1), 30 were diagnosed with mild COVID-19 without aspiration pneumonia (Group A2), and 106 were diagnosed with aspiration pneumonia alone. COVID-19 was confirmed through a positive result in the reverse transcriptase polymerase chain reaction test, and pneumonia was diagnosed by conducting various imaging tests, such as chest radiography or computed tomography, along with the assessment of clinical signs of oxygen saturation in room air. This study was approved by the Institutional Review Board of the Sahmyook Medical Center (116286-202307-HR-01; 6 July 2023). Patient consent was waived due to the retrospective nature of the study. A flowchart of the study participants is shown in Figure 1.

### 2.2. VFSS

The normal process of swallowing proceeds through the oral preparatory and propulsive stages and then advances to the pharyngeal and esophageal stages [7]. VFSS is a radiographic procedure that provides a dynamic view of swallowing. It evaluates oropharyngeal swallowing in various anatomical regions using 11 distinct factors: lip closure, bolus formation, transition time, post-swallowing residue, and aspiration [18]. Rehabilitation medicine specialists and residents participated in this study. After positioning the patient on a chair, lateral fluoroscopic imaging was performed using a fluoroscopy device (Brivo OEC 850, Milwaukee, WI, USA). First, the patients were instructed to initially swallow 2 mL of barium contrast solution. Subsequently, they were asked to swallow 5 mL of barium contrast solution. If no aspiration occurred, the patients were asked to swallow each of following thin porridge, thick porridge, and boiled rice, all of which were combined with a barium contrast solution. For patients with no swallowing dysfunction, the swallowing procedure was performed once, and then they proceeded to the next step. In cases with penetration, the procedure was repeated twice for confirmation. If aspiration occurred at any point, the study was terminated. All the processes were recorded at a rate of 30 frames per second, and the recorded videos were analyzed frame-by-frame to assess the swallowing function after completing the examination.

### 2.3. Penetration–Aspiration Scale Measurement

The penetration–aspiration scale (PAS) is an 8-point interval scale designed to assess the severity of penetration and aspiration [19]. The PAS scores are categorized as follows: normal swallowing (PAS 1), penetration above the vocal folds (PAS 2–3), contact with the vocal folds (PAS 4–5), aspiration below the vocal folds (PAS 6–7), and silent aspiration (PAS 8), with increasing scores indicating higher severity and risk of complications. In our study, each patient’s PAS score was determined by identifying the highest stage observed during VFSS. A higher PAS score indicated decreased swallowing function.

### 2.4. Functional Dysphagia Scale Measurement

The functional dysphagia scale (FDS) is a tool based on the VFSS developed to evaluate the swallowing function in patients (Table 1). 

It is a sensitive and specific method for diagnosing and assessing the severity of dysphagia [20]. Eleven distinct factors were used to evaluate the swallowing function in individual anatomical regions. The total score is 100 points, with a higher score indicating deterioration in swallowing function.

### 2.5. Statistical Analysis

Descriptive statistics, Fisher’s exact test, or Pearson’s chi-square test were used to compare the categorical data, while the Kruskal–Wallis test and Mann–Whitney U test were used for comparisons of continuous variables. Subsequent post hoc analyses were conducted to distinguish the differences among the three groups. A *p* value of <0.05 was considered significant, and all statistical analyses were performed using commercial software (IBM SPSS Statistics. Version 27.0. Armonk, NY, USA: IBM Corp.).

## 3. Results

### 3.1. Baseline Characteristics

The demographic and clinical characteristics of the study participants are presented in Table 2 and Table 3. In a comparison between patients with mild COVID-19 (Group A1 and A2) and those with aspiration pneumonia alone (Group B), no significant differences were observed in the prevalence of dysphagia-related comorbidities, such as stroke, Parkinson’s disease, dementia, asthma, and chronic obstructive pulmonary disease.

### 3.2. Comparison of VFSS Parameter by Group

**Figure 2:**
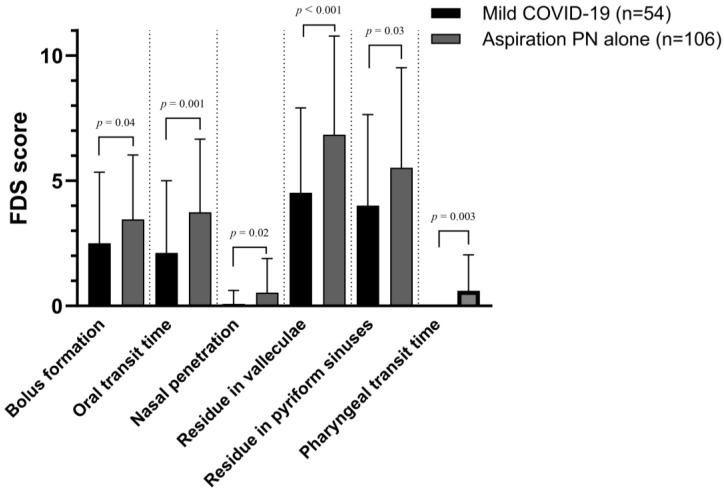
Comparison of FDS scores based on VFSS between mild COVID-19 group and aspiration PN alone group for parameters with significant difference. FDS, functional dysphagia scale; VFSS, videofluoroscopic swallowing study; PN, pneumonia.

Table 4 and Figure 2 depict the differences in the VFSS parameters between Groups A and B. Group A tended to have lower PAS and FDS scores than Group B, indicating milder dysphagia. Among the FDS sub-scores, oral transit time (*p* = 0.001) and pharyngeal transit time (*p* = 0.003) were significantly shorter in Group A. Additionally, the prevalence rates of bolus formation (*p* = 0.04), nasal penetration (*p* = 0.02), residue in the vallecula (*p* < 0.001), and residue in the pyriform sinus (*p* = 0.03) were lower in Group A (Table 4 and Figure 2).

### 3.3. Comparison of VFSS Parameters by Subgroup

**Figure 3:**
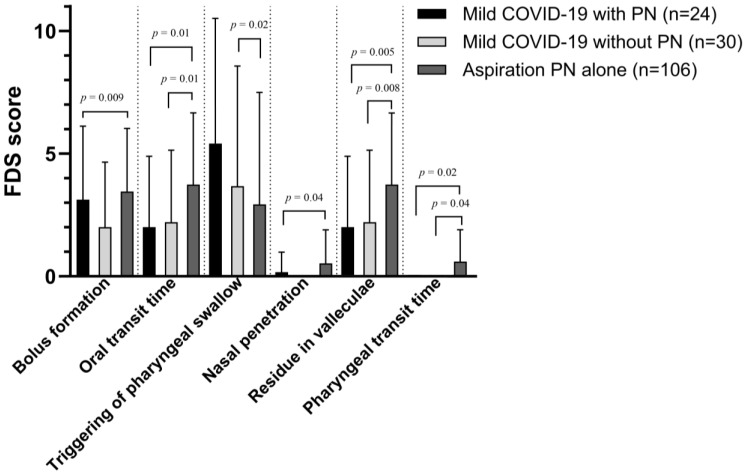
Comparison of FDS scores based on VFSS among mild COVID-19 with PN group, mild COVID-19 without PN, and aspiration pneumonia alone group for parameters with significant difference. FDS, functional dysphagia scale; VFSS, videofluoroscopic swallowing study; PN, pneumonia.

The patients were subdivided into COVID-19 with aspiration pneumonia (Group A1), COVID-19 without aspiration pneumonia (Group A2), and aspiration pneumonia alone (Group B) (Table 5 and Figure 3). The bolus formation was better in Group A1 than in Group B (*p* = 0.009). The oral transit times in Groups A1 and A2 were shorter than that in Group B (*p* = 0.01). The amount of vallecular residues was lower in Groups A1 and A2 than in Group B (*p* = 0.005 and *p* = 0.008, respectively). Similarly, the pharyngeal transit time was shorter in Groups A1 and A2 than in Group B (*p* = 0.02 and *p* = 0.04, respectively). Nasal penetration also differed between Groups A1 and B (*p* = 0.04). Group B showed improvement in the triggering of pharyngeal swallowing compared with Group A2 (*p* = 0.02).

## 4. Discussion

This study primarily aimed to identify the differences in swallowing dysfunction between patients with mild COVID-19 and those with aspiration pneumonia using VFSS. The mild COVID-19 group exhibited lower PAS and FDS scores, showing less severe dysphagia, compared with the scores in the aspiration pneumonia-only group. This trend was evident in several VFSS parameters, such as transit time and post-swallowing residue. When the mild COVID-19 group was subdivided into COVID-19 with aspiration pneumonia (Group A1) and without aspiration pneumonia (Group A2), both subgroups A1 and A2 showed better VFSS parameters than Group B, with specific differences in oral and pharyngeal transit times, vallecular residues, and nasal penetration. These findings indicate that patients with mild COVID-19 experience mild degrees of dysphagia, irrespective of the status of aspiration pneumonia, compared with those with aspiration pneumonia alone. 

According to the WHO classification, mild COVID-19 has less severe symptoms compared with severe COVID-19 [2]. Shadi et al. [17] reported that complications of COVID-19 are associated with the degree of severity. Jeon et al. [14] reported that patients with severe COVID-19 exhibited more pronounced dysphagia compared to those with aspiration pneumonia. Based on these previous studies, our research finding that mild COVID-19 is associated with milder dysphagia compared to aspiration pneumonia confirms our hypothesis.

Severe acute respiratory syndrome coronavirus 2 (SARS-CoV-2), the causative pathogen for COVID-19, demonstrates a predilection for binding to angiotensin-converting enzyme 2 (ACE2) [21], a receptor found across multiple organ systems, including neural tissues [22]. Previous studies have reported cases of cranial nerve infections following COVID-19, as well as cognitive impairment due to diffuse white matter damage [23,24]. Symptoms such as loss of smell and Bell’s palsy also result from olfactory and facial nerve involvement [25,26].

Among the cranial nerves involved in swallowing, damage to the trigeminal nerve can affect mastication because it innervates the masticatory muscles [7]. The facial nerve plays a role in preventing food leakage during swallowing by controlling lip movement [27]. Damage to the glossopharyngeal and vagus nerves can adversely affect pharyngeal swallowing [27,28]. Similarly, hypoglossal nerve damage may affect tongue movement, which can influence bolus formation [29]. This could mean that the virus directly affects the nerves responsible for swallowing, thus contributing to the emergence of dysphagia in patients with COVID-19 [30,31]. The possibility of nerve damage could also explain the delays in the triggering of pharyngeal swallowing in Group A2 compared with that in Group B. 

When the SARS-CoV-2 virus binds to the ACE2 receptors in the respiratory epithelium, it triggers an inflammatory response via a cytokine storm. This response can range from asymptomatic to severe, which may progress to ARDS [3], potentially necessitating mechanical ventilation (MV) [31]. Invasive respiratory support, such as endotracheal intubation or MV, has been associated with the increased prevalence and severity of swallowing difficulty [11,12,13,14]. Frajkova et al. [5] reported that dysphagia during intubation can be attributed to various factors, such as the duration of MV, which is linked to the incidence of dysphagia; worsening of nutritional status due to reduced food intake while on MV; and the possibility of injury to oral structures during the intubation process. Considering that MV has been linked to a higher risk of dysphagia [5,6], the fact that the mild COVID-19 group did not require invasive treatments and had a lower risk of nerve damage than the severe-to-critical group implies that their dysphagia was probably less severe.

Age was a significant factor in the occurrence of dysphagia [13,32,33]. Presbyphagia is a swallowing disorder that can occur due to aging, even in otherwise healthy individuals [34]. This is due to the weakening of swallowing-related muscles caused by sarcopenia [35]. Martín et al. [36] reported that as the average age increased, frailty became more severe, and the swallowing function declined. However, the existing literature comparing the incidence of presbyphagia and dysphagia resulting from COVID-19 is limited. Considering that the average age of Groups A1 (82.62 ± 1.41) and A2 (84.43 ± 1.73) was higher than that of Group B (81.58 ± 0.72), Groups A1 and A2 were expected to have a poorer swallowing function when considering the effects of presbyphagia. However, our observation that Groups A1 and A2 demonstrated better swallowing function might imply that the effect of mild COVID-19 on swallowing disorders is less severe than that of presbyphagia.

Sarcopenia is defined by a reduction in both the quantity and strength of skeletal muscles [37], leading to impaired respiratory and swallowing muscle functions, malnutrition, and a weakened immune system [38]. The impaired functions can increase susceptibility to COVID-19. Of note, isolation in a hospital or at home for several days following COVID-19 diagnosis, along with a decrease in activity levels due to deconditioning, promotes muscle loss and contributes to the onset of sarcopenia [39]. Sarcopenia experienced by the elderly can lead to muscle loss and subsequently trigger dysphagia, creating a vicious cycle [40]. To break this detrimental cycle, appropriate post-infection dysphagia rehabilitation is essential to facilitate functional recovery. Dysphagia emerging after COVID-19 shows rapid improvement with short-term therapy [41], and speech therapists can provide swallowing rehabilitation with several speech–language therapies and oral motor facilitation techniques [42]. However, the rehabilitation procedures were considered aerosol-generating procedures. Therefore, when evaluating and treating swallowing disorders in patients with COVID-19, healthcare professionals and patients are at risk of infection, emphasizing the importance of the thorough use of personal protective equipment [43]. 

While the WHO continues to classify the pandemic as ongoing, certain nations are shifting their public health strategies to treat COVID-19 as an endemic disease. Safety restrictions that have been impeding dysphagia treatment will gradually be loosened, allowing for more proactive treatment. Our research findings may be useful in developing treatment strategies for dysphagia accompanying mild COVID-19.

Our study has several limitations. First, owing to the retrospective nature of our study, we were unable to control the time interval between the onset of symptoms and the timing of the tests, leading to potential bias and limitations in data collection. Also, we were unable to verify the spontaneous resolution of COVID-19-induced dysphagia via follow-up evaluations. Considering the relatively early recovery of swallowing function in patients who developed dysphagia after COVID-19 [16,44], using a prospective study design could help overcome these limitations and yield more objective and precise findings. Second, the limited sample size hindered in-depth subgroup evaluations; participants were exclusively recruited from a secondary medical institute, and patients with suspected aspiration pneumonia or COVID-19, who were more likely to experience dysphagia, were exclusively included in the study. Patients without respiratory symptoms were not part of the research, which further limits the generalizability of our study findings. Third, the study failed to account for additional medical conditions which could potentially influence dysphagia. In our study, no significant differences were found between the groups in terms of the incidence of stroke or Parkinson’s disease, both of which are known to influence swallowing difficulty. However, a more detailed comparison of the dysphagia-related factors, including sex, age, and presence of diseases, such as multiple sclerosis, myopathy, and Guillain–Barré syndrome, would have been possible if neurological assessments had been included.

## 5. Conclusions

Patients with mild COVID-19 had lower PAS and FDS scores, suggesting milder dysphagia than for those with aspiration pneumonia. This pattern persisted in multiple VFSS parameters. When the patients were further divided into subgroups of COVID-19 with and without aspiration pneumonia, both subgroups outperformed the aspiration pneumonia group in specific VFSS parameters, including oral and pharyngeal transit times and vallecular residue. During the COVID-19 pandemic, there have been limitations on treatment. However, if COVID-19 changes to an endemic disease with fewer treatment limitations, proactive dysphagia rehabilitation for mild COVID-19 patients could be enabled, potentially aiding in the rapid recovery of swallowing disorders.

## Figures and Tables

**Table 1:** FDS parameters based on VFSS.

Factor	Assigned Value		Score
Lip closure	Intact	0	10
	Inadequate	5	
	None	10	
Bolus formation	Intact	0	6
	Inadequate	3	
	None	6	
Residue in oral cavity	None	0	6
	≤10%	2	
	10–50%	4	
	≥50%	6	
Oral transit time	≤1.5 s	0	6
	>1.5 s	6	
Triggering of pharyngeal swallow	Normal	0	10
	Delayed	10	
Laryngeal elevation and epiglottic closure	Normal	0	12
	Reduced	12	
Nasal penetration	None	0	12
	≤10%	4	
	10–50%	8	
	≥50%	12	
Residue in vallecula	None	0	12
	≤10%	4	
	10–50%	8	
	≥50%	12	
Residue in pyriform sinuses	None	0	12
	≤10%	4	
	10–50%	8	
	≥50%	12	
Coating of pharyngeal wall after swallow	No	0	10
	Yes	10	
Pharyngeal transit time	≤1.0 s	0	4
	>1.0 s	4	
Total			100

FDS, functional dysphagia scale; VFSS, videofluoroscopic swallowing study.

**Table 2:** Demographic characteristics of patients.

	Group A1 (*n* = 24)	Group A2 (*n* = 30)	Group B (*n* = 106)
Age (years)	82.62 ± 1.41	84.43 ± 1.73	81.58 ± 0.72
Sex			
Male	12 (50%)	11 (36.7%)	71 (67.0%)
Female	12 (50%)	19 (63.3%)	35 (33.0%)
Vaccination			
Yes	17 (70.8%)	19 (63.3%)	45 (42.4%)
No	7 (29.2%)	11 (36.7%)	61 (57.6%)
Antiviral treatment			
Yes	19 (79.2%)	13 (43.3%)	
No	5 (20.8%)	17 (56.7%)	

Continuous variables are presented as mean ± standard deviation (SD) and categorical variables as number and percentage.

**Table 3:** Comorbidities of patients with COVID-19 or aspiration pneumonia.

	Group A1 (*n* = 24)	Group A2 (*n* = 30)	Group B (*n* = 106)	*p*-Value
Stroke				0.84
No	15 (62.5%)	21 (70%)	70 (66.0%)	
Yes	9 (37.5%)	9 (30%)	36 (34.0%)	
Parkinson				0.74
No	21 (87.5%)	28 (93.3%)	97 (91.5%)	
Yes	3 (12.5%)	2 (6.7%)	9 (8.5%)	
Dementia				0.31
No	14 (58.3%)	19 (63.3%)	77 (72.6%)	
Yes	10 (41.7%)	11 (36.7%)	29 (27.4%)	
Asthma				0.37
No	23 (95.8%)	26 (86.7%)	99 (93.4%)	
Yes	1 (4.2%)	4 (13.3%)	7 (6.6%)	
COPD				0.26
No	22 (91.7%)	30 (100%)	97 (91.5%)	
Yes	2 (8.3%)	0 (0%)	9 (8.5%)	

Categorical variables are presented as number and percentage. Variables were compared using Fisher’s exact test or Pearson’s chi-square test as appropriate. COPD, chronic obstructive pulmonary disease; COVID-19, coronavirus disease.

**Table 4:** Comparison of PAS and FDS scores based on VFSS between Groups A and B.

	Group A (*n* = 54)	Group B (*n* = 106)	*p*-Value
PAS	4.31 (2.73)	4.88 (2.83)	0.21
Lip closure	1.02 (2.975)	0.57 (1.87)	0.64
Bolus formation	2.5 (2.84)	3.45 (2.58)	**0.04**
Residue in oral cavity	1.74 (1.456)	2.15 (1.87)	0.23
Oral transit time	2.11 (2.892)	3.74 (2.92)	**0.001**
Triggering of pharyngeal swallow	4.44 (5.016)	2.92 (4.57)	0.06
Laryngeal elevation and epiglottic closure	6.44 (6.04)	4.87 (5.92)	0.12
Nasal penetration	0.07 (0.544)	0.53 (1.36)	**0.02**
Residue in vallecula	4.52 (3.391)	6.83 (3.94)	**<0.001**
Residue in pyriform sinuses	4 (3.645)	5.51 (4)	**0.03**
Coating of pharyngeal wall after swallow	4.81 (5.043)	5.38 (5.01)	0.50
Pharyngeal transit time	0 (0)	0.6 (1.44)	**0.003**
Total	31.67(21.466)	36.55(21.39)	0.20

Continuous variables are presented as mean ± standard deviation (SD). Bold numbers depict significant difference (*p*-value < 0.05). FDS, functional dysphagia scale; PAS, penetration–aspiration scale; VFSS, videofluoroscopic swallowing study.

**Table 5:** Comparison of PAS and FDS scores based on VFSS among Groups A1, A2, and B.

	Group A1 (*n* = 24)	Group A2 (*n* = 30)	Group B (*n* = 106)	*p*-Value	Group 1–2	Group 2–3	Group 1–3
PAS	3.90 (2.64)	4.83 (2.79)	4.88 (2.83)	0.23	0.22	0.86	0.09
Lip closure	1.04 (2.94)	1 (3.05)	0.57 (1.87)	0.87	0.81	0.60	0.83
Bolus formation	3.13 (3)	2 (2.65)	3.45 (2.58)	**0.04**	0.17	0.66	**0.009**
Residue in oral cavity	1.67 (1.4)	1.8 (1.52)	2.15 (1.87)	0.47	0.81	0.3	0.4
Oral transit time	2 (2.89)	2.2 (2.94)	3.74 (2.92)	**0.005**	0.80	**0.01**	**0.01**
Triggering of pharyngeal swallow	5.42 (5.09)	3.67 (4.9)	2.92 (4.57)	0.07	0.20	**0.02**	0.439
Laryngeal elevation and epiglottic closure	6 (6.13)	6.8 (6.05)	4.87 (5.92)	0.26	0.63	0.4	0.118
Nasal penetration	0.17 (0.82)	0 (0)	0.53 (1.36)	0.06	0.26	0.212	**0.04**
Residue in vallecula	4.5 (3.19)	4.53 (3.6)	6.83 (3.94)	**0.002**	0.99	**0.008**	**0.005**
Residue in pyriform sinuses	4 (3.54)	4 (3.79)	5.51 (4)	0.09	0.94	0.10	0.07
Coating of pharyngeal wall after swallow	5 (5.11)	4.67 (5.07)	5.38 (5.01)	0.78	0.81	0.74	0.49
Pharyngeal transit time	0 (0)	0 (0)	0.6 (1.44)	**0.01**	1	**0.04**	**0.02**
Total	32.92(22)	30.67(21.35)	36.55(21.39)	0.42	0.72	0.46	0.23

Kruskal–Wallis test was used to compare the three groups, while Mann–Whitney U test was used to compare two groups. Continuous variables are presented as mean ± standard deviation (SD). Bold numbers depict significant difference (*p*-value < 0.05). FDS, functional dysphagia scale; PAS, penetration–aspiration scale; VFSS, videofluoroscopic swallowing study.

## Data Availability

The data used and/or analyzed during the current study are available from the corresponding author upon reasonable request.
